# Differential interactions between IGFBP-3 and transforming growth factor-beta (TGF-β) in normal *vs* cancerous breast epithelial cells

**DOI:** 10.1038/sj.bjc.6600355

**Published:** 2002-06-17

**Authors:** C McCaig, C A Fowler, N J Laurence, T Lai, P B Savage, J M P Holly, C M Perks

**Affiliations:** Division of Surgery, Department of Hospital Medicine, Bristol Royal Infirmary, Bristol BS2 8HW, UK

**Keywords:** insulin-like growth factor-binding protein-3 (IGFBP-3), cancer, normal, TGF-β, apoptosis, proliferation

## Abstract

In addition to modulating insulin-like growth factors action, it is now clear that insulin-like growth factor-binding protein-3 also has intrinsic effects on cell growth and survival. We have compared the effects of insulin-like growth factor-binding protein-3 and transforming growth factor-beta on cell proliferation and death of Hs578T cells and the normal breast epithelial cell line, MCF-10A. The growth of MCF-10A cells was inhibited at low concentrations of insulin-like growth factor-binding protein-3 but stimulated at high concentrations. These differential effects were unaffected in the presence of an insulin-like growth factor-I receptor antagonist. A synthetic peptide corresponding to the serine phosphorylation domain of insulin-like growth factor-binding protein-3 (that does not bind to insulin-like growth factors) also mimicked these differential actions. The growth of both cell lines was significantly inhibited by transforming growth factor-beta, this was associated with a 14-fold increase of insulin-like growth factor-binding protein-3 secreted by the Hs578T cells but a five-fold decrease of insulin-like growth factor-binding protein-3 secreted by MCF-10A cells. Replacement doses of exogenous insulin-like growth factor-binding protein-3 overcame the transforming growth factor-beta-induced growth inhibition in the MCF-10A cells. Cell death induced by ceramide was significantly reduced by insulin-like growth factor-binding protein-3 in the MCF-10A cells and depleting insulin-like growth factor-binding protein-3 with transforming growth factor-beta in these cells consequently increased their susceptibility to ceramide. In contrast, insulin-like growth factor-binding protein-3 enhanced apoptosis induced by ceramide in the Hs578T cells but transforming growth factor-beta treated Hs578T cells were resistant to apoptosis. The addition of anti-sense mRNA to insulin-like growth factor-binding protein-3 significantly abrogated this effect of transforming growth factor-beta. These data indicate that insulin-like growth factor-binding protein-3 has intrinsic activity capable of inhibiting or enhancing the growth and survival of breast epithelial cells depending on the cell line and exposure to other cytokines.

*British Journal of Cancer* (2002) **86**, 1963–1969. doi:10.1038/sj.bjc.6600355 www.bjcancer.com

© 2002 Cancer Research UK

## 

The insulin-like growth factors (IGF)-I and -II are potent mitogens acting through the IGF-I tyrosine kinase receptor. The high affinity IGF-binding proteins (IGFBP) modulate the availability of the IGFs to interact with their receptor. In the circulation, the IGFs are carried mainly by IGFBP-3, which together with the acid labile subunit (ALS) forms a 150 KDa complex. This complex serves to prolong the half-life of the IGFs (Jones and Clemmons, 1995). In addition to the IGF-dependent functions, it is becoming increasingly clear that the IGFBPs also possess IGF-independent activity. In the IGF unresponsive breast cancer cell line, Hs578T and the IGF-responsive normal breast epithelial cell line, MCF-10A, IGFBP-3 can inhibit cell growth (Oh *et al*, 1993a; Martin and Baxter, 1999). It has also been reported that IGFBP-3 alone can induce apoptosis in prostate cancer cells (Rajah *et al*, 1997). We have demonstrated previously that IGFBP-3 did not induce apoptosis alone but could accentuate cell death induced by triggers of apoptosis such as a ceramide analogue (C2) (Gill *et al*, 1997), Antimycin A (Perks *et al*, 2000a), UV radiation (Hollowood *et al*, 2000a), gamma radiation (Williams *et al*, 2000) and paclitaxel (Fowler *et al*, 2000), but had no effect on cell death induced by an arg-gly-asp (RGD)-containing disintegrin (Perks *et al*, 1999). The mechanism by which IGFBP-3 can induce these IGF-independent actions is still unclear. There have been reports of a putative IGFBP-3 receptor (Oh *et al*, 1993b), and that IGFBP-3 can interact with mitogen activated protein kinase (MAP kinase) signalling (Martin and Baxter, 1999). We demonstrated previously that treatment with IGFBP-3 resulted in a rapid dephosphorylation of focal adhesion kinase (FAK) that associates with activated integrin receptors (Perks and Holly, 1999) as well as inhibiting cell adhesion of Hs578T cells to an extracellular matrix (Perks *et al*, 2000b).

Transforming growth factor-beta (TGF-β) is a cytokine that regulates cell growth, differentiation, morphogenesis and apoptosis. TGF-β is a growth stimulator of fibroblast cells but is growth inhibitory in breast epithelial cells and mammary tumours (Daniel *et al*, 1996; Gold, 1999). Growth inhibitors such as TGF-β, retinoic acid and vitamin D (Colston *et al*, 1998) are potent regulators of IGFBP-3 and can stimulate IGFBP-3 at the mRNA level. TGF-β-induced growth inhibition seen in Hs578T breast cancer cells, as well as the TGF-β-induced proliferation seen in colon cancer cells and human airway smooth muscle cells is at least partially mediated by IGFBP-3 (Oh *et al*, 1995; Cohen *et al*, 2000; Kansra *et al*, 2000). The relationship between IGFBP-3 and TGF-β also extends to the receptors and the signalling pathways. There have been reports that the type V TGF-β receptor maybe a putative IGFBP-3 receptor (Leal *et al*, 1997) and that IGFBP-3 may interact with the SMAD signalling proteins and the type II TGF-β receptor to induce its growth inhibitory actions (Fanayan *et al*, 2000).

In this study we looked at the effects of IGFBP-3 on cell growth and death in the normal breast epithelial cell line, MCF-10A and compared the effects of TGF-β on the growth, death and the production of IGFBP-3 in these cells to that in the Hs578T breast cancer cell line.

## MATERIALS AND METHODS

### Materials

Recombinant human non-glycosylated IGFBP-3 (ngIGFBP-3) was a kind gift from Dr C Maack (Celtrix, CA, USA). Human recombinant IGF-I was purchased from Gropep (Adelaide, Australia). The ceramide analogue, C2, was purchased from Calbiochem (Nottingham, UK). The IGF-1 receptor antagonist (IGFIR-AT) was purchased from Immunological and Biochemical Testsystems (IBT) GmbH (Reutlingen, Germany). The IGFIR-AT is an IGF-I peptide analogue that acts as a competitive ligand to block receptor autophosphorylation. It has been shown to inhibit the proliferation of a number of cell lines in a dose-dependent manner. This peptide JB1 was prepared according to the sequence described by Pietrzkowski *et al* (1992): H-CYAAPLKPAKSC-NH_2_. In the normal MCF-10A cells, the concentration of the IGFIR-AT used (100 ng ml^−1^) did not have any effect on basal, unstimulated cell proliferation whereas concentrations of 120 ng ml^−1^ and above inhibited cell proliferation (data not shown). Human recombinant transforming growth factor-beta1 (TGF-β) and all other chemicals were purchased from Sigma (Poole, UK). Tissue culture plastics were obtained from Greiner Labortechnik Ltd (Stonehouse, UK). Serine phosphorylation domain (SPD) peptide is a 15 amino acid peptide sequence spanning the two mid-region serines of IGFBP-3. It was synthesised at the microchemical facility of the Babraham Institute (Cambridge, UK). We have demonstrated previously that of 17 peptides corresponding to different regions of IGFBP-3, SPD was the only one which was able to mimic the actions observed with full length IGFBP-3 on cell death. In addition SPD had no interaction with IGF-I (Hollowood *et al*, 2000b). The IGFBP-3 antisense oligodeoxynucleotide was prepared by the Department of Biochemistry, University of Bristol (Bristol, UK). The IGFBP-3 antisense mRNA was complementary to 20 nucleotides that encode the N-terminus of human IGFBP-3 and had the sequence 5′-CAT GAC GCC TGC AAC CGG GG-3′ (positions 2021–2040); the sequence of the sense mRNA was 5′-CCC CGG TTG CAG GCG TCA TG-3′.

### Cell culture

Human breast cancer cells, Hs578T were purchased from ECACC (Porton Down, Wiltshire, UK) and grown in humidified 5% carbon dioxide atmosphere at 37°C. The cells were cultured in Dulbecco's modified Eagles Medium (DMEM) with glutamax-1 supplemented with 10% foetal calf serum (FCS), penicillin (500 iu ml^−1^), streptomycin (5 mg ml^−1^) and L-glutamine (2 nM) –growth media (GM). The MCF-10A cells are a spontaneously immortalised breast epithelial cell line that maintain a relatively normal phenotype in that they (a) lack tumourgenicity in nude mice, (b) exhibit 3D growth in collagen, (c) their growth was controlled by hormones and growth factors and (d) they form domes in confluent cultures (Soule *et al*, 1990). These cells were purchased from ATCC (Manassas, VA, USA) and grown in humidified 5% carbon dioxide atmosphere at 37°C. The cells were cultured in a 1 : 1 mixture of DMEM : Hams Nutrient Mix F12 media supplemented with 5% horse serum (HS), penicillin (500 iu ml^−1^), streptomycin (5 mg ml^−1^), L-glutamine (2 nM), cholera toxin (100 ng ml^−1^), insulin (10 μg ml^−1^), EGF (20 ng ml^−1^) and hydrocortisol (0.5 μg ml^−1^). Experiments for both cell lines were performed in phenol red-and serum-free 1 : 1 mixture of DMEM : Hams Nutrient Mix F12 supplement with sodium bicarbonate (0.12%), bovine serum albumin (BSA) (0.2 mg ml^−1^), transferrin (0.1 mg ml^−1^), penicillin (500 iu ml^−1^), streptomycin (5 mg ml^−1^) and L-glutamine (2 nM) (SFM).

### Cell dosing protocol

The MCF-10A cells were seeded in six well plates and grown in GM for 24 h. Cells were either (a) switched to SFM for 48 h before dosing with ngIGFBP-3 or SPD for 24 h, (b) switched to SFM for 24 h before dosing with TGF-β for 3 days with a co-incubation with an apoptotic dose of C2 ceramide (25–35 μM) on the penultimate day; (c) switched to SFM for 24 h before dosing with TGF-β and ngIGFBP-3 as described in figure legends; or (d) switched to SFM for 24 h prior to a pre-incubation with ngIGFBP-3 for 24 h followed by a co-incubation with an apoptotic dose of C2-ceramide.

The Hs578T breast cancer cells were seeded in six well plates in GM for 24 h prior to switching to SFM for a further 24 h. Cells were: (a) pre-incubated with IGFBP-3 (100 ng ml^−1^) for 24 h followed by a co-incubation with an apoptotic dose of C2; or (b) dosed with TGF-β in low serum (2%) media for 5 days with or without 20 μg ml^−1^ anti-sense or sense mRNA to IGFBP-3 on days 1, 3 and 5 followed by a co-incubation on the penultimate day with an apoptotic dose of C2-ceramide (20–30 μM). The final dose used in each experiment was dependent on the confluency and passage number of the cells.

### Trypan blue dye exclusion

Aliquots of cells were loaded onto a haemocytometer (1 : 1) with Trypan blue. Viable cells exclude the dye. Both living and dead cells were counted, from which the percentage dead cells or percentage of cells relative to the control were calculated.

### Flow cytometry

This technique was used to determine the amount of apoptosis in any given sample. The fragmented DNA of an apoptotic cell has less capacity to stain than in normal cells and appears as a pre-G1 peak on a DNA cell cycle histogram. Cells (1–2×10^6^) were washed in phosphate buffered saline (PBS) and fixed in 70% ethanol for a minimum of 30 min prior to analysis. The fixed cells were pelleted (6500 r.p.m., 5 min) and washed in PBS. The cells were resuspended in 500 μl of reaction buffer (propidium iodide, 0.05 mg ml^−1^; sodium citrate, 0.1%; RNase A, 0.02 mg ml^−1^; NP-40, 0.3% pH 8.3). This was incubated for 30 min at 4°C prior to measurement using a FACS Calibur Flow Cytometer (Becton Dickinson, Plymouth, UK) with an argon laser at 488 nm for excitation. Analysis was by Cell Quest software package (Becton Dickinson).

### Radioimmunoassay

Levels of IGFBP-3 in conditioned media were measured by radioimmunoassay as previously described (Yateman *et al*, 1993).

The basal levels of IGF-I and -II produced by both the MCF-10A and the Hs578T cells, as measured by radioimmunoassays previously described (Taylor *et al*, 1990; Davies *et al*, 1991) were found to be lower than the detection limits of the assays and hence negligible in relation to the doses required for IGF responses in these cells (i.e. <1 ng ml^−1^; data not shown).

### Statistical analysis

The data were analysed using Microsoft Excel 2000 software package. Significant differences were determined using Students *t*-test. Statistically significant differences were considered to be present at *P*<0.05. All graphs represent the mean of experiments that were each performed in triplicate at least three times±standard error of the mean.

## RESULTS

### Differential, IGF-independent effects of IGFBP-3 on growth of MCF-10A cells

**Figure 1:**
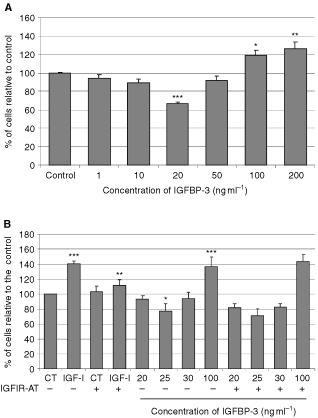
Differential, IGF-independent effects of IGFBP-3 on growth of MCF-10A cells. Graphs represent the per cent of cells relative to control. Cells were treated (**A**) with increasing doses of ngIGFBP-3 for 24 h (where *P*<0.05*; *P*<0.01**; *P*<0.001*** relative to the control (CT)), or (**B**) with IGF-1 (100 ng ml^−1^) or IGFBP-3 with or without the IGFIR-AT (where ***IGF-I>CT and IGFBP-3 (100 ng ml^−1^)>CT; *P*<0.001, **IGF-I+IGFIR-AT<IGF-I; *P*<0.01, *IGFBP-3 (20 ng ml^−1^)<CT; *P*<0.05). Graph represents the mean of experiments that were each performed in triplicate at least three times.

The addition of increasing doses of IGFBP-3 to the MCF-10A cells resulted in a biphasic response with inhibition of growth at concentrations of 20 ng ml^−1^ (*P*<0.001), but enhanced growth at higher doses of 100 and 200 ng ml^−1^ (*P*<0.01) (Figure 1A

).

To determine whether these effects of IGFBP-3 were IGF-I independent we used an IGF-I receptor antagonist (Figure 1B). At 100 ng ml^−1^, IGF-I significantly (*P*<0.001) increased basal cell number by 41%. The IGF-I receptor antagonist alone had no effect but on co-incubation with IGF-I, the increase in cell number was decreased from 141 to 112% (*P*<0.01). As before IGFBP-3 at low doses significantly (*P*<0.05) decreased cell number by 27.8% and at 100 ng ml^−1^, significantly (*P*<0.001) increased cell number by 37%. These effects of IGFBP-3 were identical in the presence of the IGF-I receptor antagonist.

### The effects of the SPD peptide on growth of MCF-10A cells

**Figure 2:**
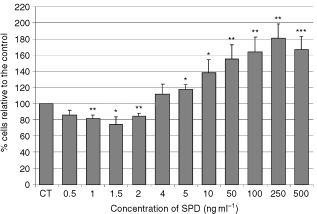
The effects of the SPD peptide on growth of MCF-10A cells. Graphs represent per cent of cells relative to the control. Cells were treated with increasing doses of SPD for 24 h where *P*<0.05*; *P*<0.01**; *P*<0.001*** relative to control (CT). Graph represents the mean of experiments that were each performed in triplicate at least three times.

The SPD peptide mimicked the biphasic effect that IGFBP-3 exerted on the growth of MCF-10A cells; with inhibition of growth at concentrations of 1.5 ng ml^−1^ (*P*<0.05; molar equivalent to 30 ng ml^−1^ IGFBP-3), but enhanced growth at concentrations of 5 ng ml^−1^ and above (*P*<0.01; molar equivalent to 100 ng ml^−1^ and above IGFBP-3) (Figure 2

).

### The effects of TGF-β on MCF-10A cell growth and IGFBP-3 production

**Figure 3:**
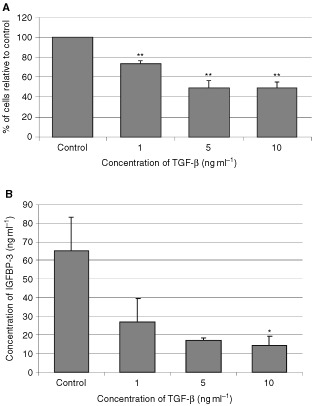
The effects of TGF-β on MCF-I0A cell growth and IGFBP-3 production. Graphs represent (**A**) the per cent of cells relative to the control and (**B**) total concentration of IGFBP-3 (ng ml^−1^). MCF-10A cells were treated with increasing doses of TGF-β for 3 days where *P*<0.01** relative to control (CT). Conditioned media was collected and IGFBP-3 levels were measures using RIA where *P*<0.05* relative to control. Graph represents the mean of experiments that were each performed in triplicate at least three times.

On MCF-10A cells (Figure 3A

), TGF-β dose-dependently inhibited cell growth with a maximum decrease in cell number of 48.5% achieved by 5 ng ml^−1^ (*P*<0.01).

Increasing doses of TGF-β induced a significant (*P*<0.05) dose-dependent decrease in the production of IGFBP-3 with a maximum decrease of 77% occurring at 10 ng ml^−1^.

### The effects of TGF-β on Hs578T cell growth and IGFBP-3 production

**Figure 4:**
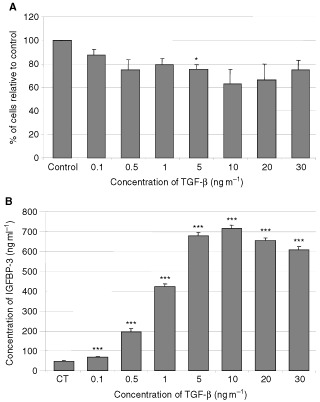
The effects of TGF-β on Hs578T cell growth and IGFBP-3 production. Graphs represents (**A**) the per cent of cells relative to the control and (**B**) the total concentration of IGFBP-3 (ng ml^−1^). Hs578T cells were treated with increasing doses of TGF-β in low serum media for 5 days (where *P*<0.05*; *P*<0.001*** relative to control). Conditioned media was collected and IGFBP-3 levels were measures using RIA. Graph represents the mean of experiments that were each performed in triplicate at least three times.

On Hs578T breast cancer cells, at concentrations of TGF-β of 5 ng ml^−1^ and above, there was a significant (*P*<0.05) decrease in cell growth compared to the control (Figure 4A

).

Increasing doses of TGF-β induced a significant (*P*<0.001 in all cases) dose-dependent increase in the production of IGFBP-3 with a maximum 14-fold increase achieved at 10 ng ml^−1^ (Figure 4B).

### The addition of exogenous IGFBP-3 overcomes TGF-β-induced growth inhibition of MCF-10A cells

**Figure 5:**
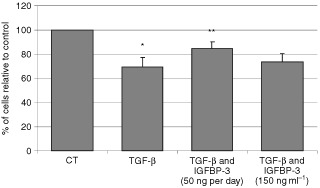
The addition of exogenous IGFBP-3 overcomes TGF-β-induced growth inhibition of MCF-10A cells. Graph shows the per cent of cells relative to the control. MCF-10A cells were treated with TGF-β (5 ng ml^−1^) for 3 days with or without IGFBP-3 (50 ng ml^−1^) added on each of the 3 days or IGFBP-3 (150 ng ml^−1^) added only on day 1 with TGF-β, (where **IGFBP-3 (50 ng day^−1^)+TGF-β>TGF-β; *P*<0.01, *TGF-β<CT; *P*<0.05). Graph represents the mean of experiments that were each performed in triplicate at least three times.

Initial experiments, showed that the total amount of IGFBP-3 produced by MCF-10A cells was 150 ng ml^−1^ after 3 days. Based on this, exogenous IGFBP-3 was added to replace the TGF-β-deleted IGFBP-3. Exposure to TGF-β (5 ng ml^−1^) significantly (*P*<0.05) inhibited cell growth by 30.7% after treatment for 3 days (Figure 5

). This inhibition of cell growth by TGF-β was significantly (*P*<0.01) abrogated when IGFBP-3 was added at 50 ng per day. When 150 ng of IGFBP-3 were added on day one, the inhibition by TGF-β was insignificantly reduced, but the daily addition of IGFBP-3 at 50 ng day was clearly more effective.

### The effects of IGFBP-3 and TGF-β on ceramide-induced death of the MCF-10A cells

**Figure 6:**
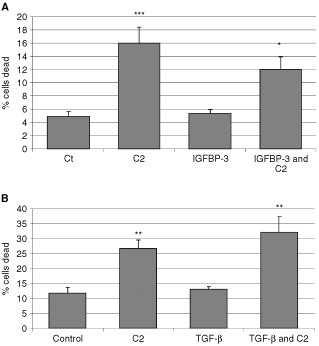
The effects of IGFBP-3 and TGF-β on ceramide-induced death of the MCF-10A cells. Graphs (**A** + **B**) represent the per cent of dead cells. MCF-10A cells were (**A**) pre-incubated with IGFBP-3 (100 ng ml^−1^) for 24 h followed by a co-incubation with an apoptotic dose of C2 for a further 24 h (where ***C2>CT; *P*<0.001, *IGFBP-3+C2<C2; *P*<0.05) or (**B**) treated with TGF-β (5 ng ml^−1^) for 3 days with or without spiking with an apoptotic dose of C2 on the penultimate day (where **C2>CT; *P*<0.01, *TGF-β+C2>C2; *P*<0.05). Graph represents the mean of experiments that were each performed in triplicate at least three times.

Ceramide significantly (*P*<0.001) increased cell death compared to control, while IGFBP-3 alone had no effect. Co-incubation of IGFBP-3 and C2 significantly (*P*<0.05) decreased cell death compared to C2 alone (Figure 6A

). Identical results were generated using flow cytometry (data not shown).

We also examined the effects of pre-treating the MCF-10A cells with TGF-β for 3 days before adding C2 on the penultimate day. Compared to basal levels, TGF-β (Figure 6B) had no effect on cell death, while the ceramide analogue (C2) significantly (*P*<0.01) increased cell death from 12 to 26.7%. The addition of C2 to cells pre-treated with TGF-β significantly (*P*<0.01) increased cell death further to 32.2% in comparison to C2 alone. Identical results were generated using flow cytometry (data not shown).

### The effect of TGF-β on ceramide-induced death of Hs578T breast cancer cells

**Figure 7:**
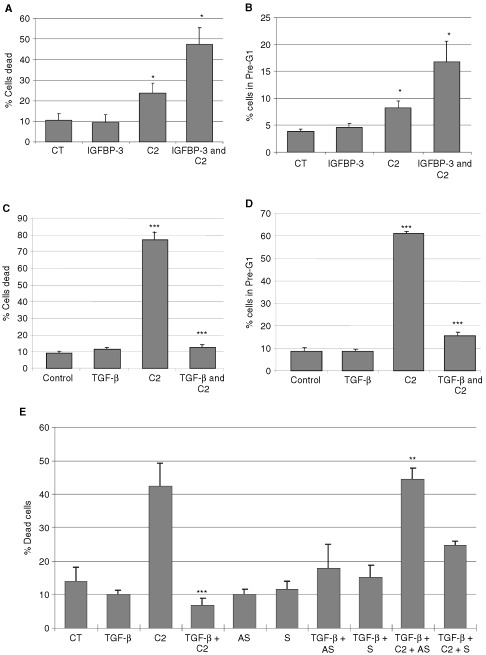
The effect of TGF-β on ceramide-induced death of Hs578T breast cancer cells. Graphs in (**A**, **C** and **E**) represent per cent dead cells while (**B**) and (**D**) represents per cent of cell in pre-G1. Where (**A**) and (**B**) Hs578T cells were pre-incubated with IGFBP-3 (100 ng ml^−1^) for 24 h followed by a co-incubation of IGFBP-3 and C2 (where C2>CT; *P*<0.05 and IGFBP-3+C2>C2; *P*<0.05). (**C** and **D**) Cells treated with TGF-β in low serum media for 4 days before an apoptotic dose of ceramide was applied (where C2>CT; *P*<0.001, TGF-β+C2<C2; *P*<0.001 in both cases) or (**E**) Cells treated with TGF-β for 5 days in low serum with or without 20 μg ml^−1^ antisense (AS) or sense (S) mRNA to IGFBP-3 added on days 1, 3, and 5 followed by a co-incubation of an apoptotic dose of ceramide on day 5, (where ***C2+TGF-β<C2; *P*<0.001, **TGF-β+C2+Antisense>TGF-β+C2; *P*<0.01). Graph represents the mean of experiments that were each performed in triplicate at least three times.

Co-incubation of C2 and IGFBP-3 significantly (*P*<0.05) increased cell death compared to C2 alone (Figure 7A

). Using flow cytometry to assess apoptosis generated identical results (Figure 7B). Death of the Hs578T cells was unaffected by 5 ng ml^−1^ TGF-β (Figure 7C). Cell death was significantly (*P*<0.001) increased to 70.9 from 9.2% by C2. Co-incubation of cells with TGF-β and C2 significantly (*P*<0.001) inhibited cell death induced by C2 from 70.9 to 12.4%. Using flow cytometry to assess apoptosis generated identical results (Figure 7D).

In Figure 7E antisense mRNA to IGFBP-3 was employed to determine if the protective effect of TGF-β against apoptosis was mediated by IGFBP-3. Ceramide increased cell death to 42.3% compared to control levels of 13.9%, while TGF-β alone had no effect. The co-incubation of TGF-β and C2 resulted in a significant (*P*<0.001) decrease in cell death to 6.8% compared to C2 alone. The sense and antisense mRNA to IGFBP-3 each alone had no effect. The survival effect conferred by TGF-β on C2-induced death was completely abrogated on co-incubation with the antisense mRNA to IGFBP-3 (*P*<0.01). The addition of TGF-β enhanced the secretion of IGFBP-3 into the conditioned media from 192 ng ml^−1^ (untreated cells) to 897 ng ml^−1^. The presence of the IGFBP-3 antisense mRNA reduced this to 327 ng ml^−1^, although there was also some suppressive effect of the sense mRNA with IGFBP-3 levels at 527 ng ml^−1^ (data not shown) and this appeared to correspond to a small increase in cell death with the sense mRNA (Figure 7E).

## DISCUSSION

In addition to being key modulators of IGF-actions, accumulating evidence suggests that the IGFBPs play an important role in the regulation of both cell growth and death, independently of their interaction with the IGFs. Growth inhibition by IGFBP-3 has been reported in many cell lines such as Hs578T and MCF-7 breast cancer cells (Colston *et al*, 1998; Oh *et al*, 1993a). In the normal breast epithelial cell line, MCF-10A, it was recently reported that IGFBP-3 at 30 ng ml^−1^ also induced growth inhibition (Martin and Baxter, 1999). In other cell lines such as human airway smooth muscles cells and colon cancer cells, IGFBP-3 has been shown to act as a potent proliferative agent (Cohen *et al*, 2000; Kansra *et al*, 2000). We determined that IGFBP-3 has differential effects on cell growth in the MCF-10A cell line. We confirmed that IGFBP-3 did inhibit cell growth at low concentrations but further determined that at higher concentrations IGFBP-3 actually promoted cell growth. These actions of IGFBP-3 were IGF-independent as the presence of an IGF-I receptor antagonist had no effect and the SPD synthetic IGFBP-3 fragment (which does not bind to IGFs) was also able to mimic the differential effects on cell growth exhibited by the intact IGFBP-3. The biphasic response of MCF-10A cells to IGFBP-3 is interesting in relation to potential mechanisms of action, but *in vivo* the prevailing high (microgram) levels of IGFBP-3 would imply that only the growth promoting action will be relevant.

In the Hs578T cell line, it has been shown that TGF-β-induced growth inhibition occurs at least partly through the increased production of IGFBP-3 (Oh *et al*, 1995). We also confirmed that TGF-β-induced growth inhibition of Hs578T cells was accompanied by an increase in IGFBP-3 production. We further determined that TGF-β also induced growth inhibition in the MCF-10A cells, but in contrast to the Hs578T cells, this was accompanied by a decrease in the levels of IGFBP-3. The addition of exogenous ngIGFBP-3 to MCF-10A cells partially overcame this growth inhibitory effect of TGF-β. This suggested that the growth inhibition induced by TGF-β in the MCF-10A cells was at least partially mediated via a decrease in the production of IGFBP-3 and further confirmed that IGFBP-3 can act as a potent proliferative agent in these normal breast epithelial cells. In addition to the opposite actions of IGFBP-3 on these two cells lines, there appears to be a big difference in sensitivity to IGFBP-3. A similar degree of growth inhibition over the same dose range of TGF-β was seen with a change in IGFBP-3 concentration of around 50 ng ml^−1^ in the MCF-10A cells but a change of around 600 ng ml^−1^ in the Hs578T cells.

We have demonstrated previously that IGFBP-3 alone, in the Hs578T cells, had no effect on cell death but accentuated C2-induced apoptosis (Gill *et al*, 1997). In the MCF-10A cells IGFBP-3 alone had no effect on cell death, but in direct contrast, it conferred survival and reduced C2-induced apoptosis. Furthermore by depleting IGFBP-3 with TGF-β in the MCF-10A cells, we found that cells were more susceptible to ceramide-induced cell death. These data are consistent with IGFBP-3 acting as a survival agent in this cell line.

We also investigated the effects of TGF-β-induced IGFBP-3 on C2-induced death in the Hs578T breast cancer cells. In contrast to adding IGFBP-3, we found that as opposed to accentuating, addition of TGF-β negated C2-induced cell death. These data reveal that IGFBP-3 can have differential effects on cell death in the Hs578T cells. We have previously shown that IGFBPs have differential effects on cell attachment depending upon the extracellular matrix (ECM) component to which the cells are exposed. While IGFBP-5 could enhance cell attachment to a general ECM, it reduced attachment to fibronectin (McCaig *et al*, 2001). It is well documented that TGF-β enhances the production of ECM components such as fibronectin (Reiss and Barcellos-Hoff, 1997) and it is attractive to speculate that the reversal of the effects of IGFBP-3 on cell survival following TGF-β pre-treatment, may similarly be related to an alteration in the compliment of ECM components. A fundamental difference between breast tumour cells and normal breast epithelial cells is the acquisition of anchorage independence; the transformed cell becomes less dependent upon the cues from the tissue structural microenvironment (Schmeichel *et al*, 1998; Chrenek *et al*, 2001). It is again attractive to speculate that the different effects of IGFBP-3 on these two breast epithelial cell lines may be related to differences in their dependence upon ECM-integrin induced intracellular signals. This clearly warrants further investigation.

**Figure 8:**
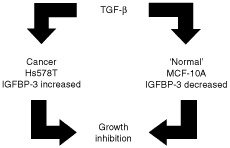
The effects of TGF-β on the production of IGFBP-3 in the normal breast epithelial cell line, MCF-10A and in the Hs578T breast cancer cell line in order to achieve growth inhibition.

In conclusion we have demonstrated that IGFBP-3 can exert differential effects on normal versus breast cancer epithelial cells. In the normal breast epithelial cells, IGFBP-3 is a potent survival factor and proliferative agent, while in the cancer cells, IGFBP-3 is an inhibitor of cell growth and accentuates cell death triggered by various agents. Consequently TGF-β-induced growth inhibition in Hs578T and MCF-10A cells is facilitated in a different way by up-regulation or down-regulation of IGFBP-3 respectively (Figure 8

).
